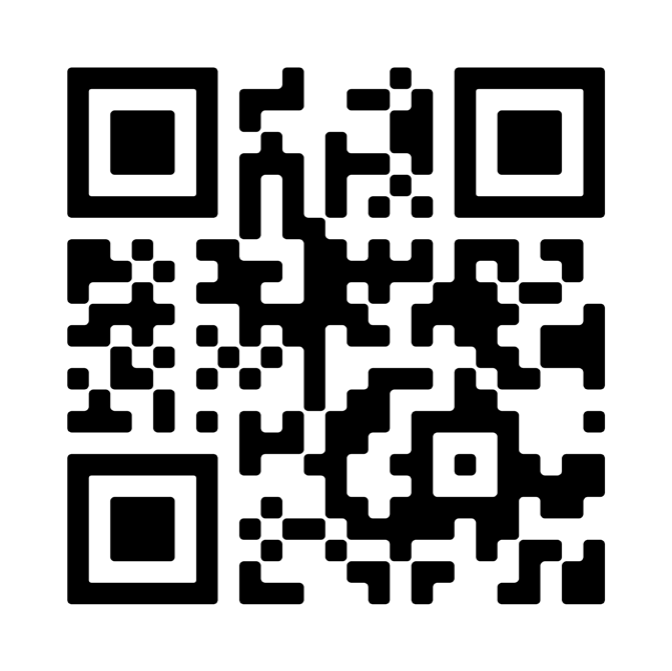# Laparoscopic repair of an anterior perineal hernia: a video presentation

**DOI:** 10.52054/FVVO.15.2.068

**Published:** 2023-06-30

**Authors:** I Furuie, A Quevedo, P Tovar, F Santos, M Zomer, G Castro, W Kondo

**Affiliations:** Department of Gynecology and Minimally Invasive Unit, Vita Batel Hospital, Curitiba, 80420-160, Brazil; Division of Minimally Invasive Gynecologic Surgery, Department of Obstetrics and Gynecology, University of Florida College of Medicine, Gainesville, 32610, United States; Department of General Surgery, Sugisawa Medical Centre, Curitiba, 80250-190, Brazil

**Keywords:** Perineal hernia, vulvar hernia, labial hernia, laparoscopic repair, laparoscopy

## Abstract

**Background:**

The perineal hernia is a condition that occurs as a result of a defect in the pelvic diaphragm. It is classified as anterior or posterior, and as either a primary or secondary hernia. The best management of this condition remains controversial.

**Objectives:**

To demonstrate the surgical steps of a laparoscopic repair with mesh of a perineal hernia.

**Materials and Methods:**

A video presentation showing the laparoscopic repair of a recurrent perineal hernia

**Main outcome measures:**

A 46-year-old woman with a prior history of a primary perineal hernia repair had complaints of a symptomatic vulvar bulge. Pelvic magnetic resonance imaging showed a 5 cm hernia sac at the right anterior pelvic wall containing adipose tissue. A laparoscopic perineal hernia repair was performed by dissection of the space of Retzius, reduction of the hernial sac, closure of the defect and mesh fixation.

**Results:**

The laparoscopic repair with mesh of a recurrent perineal hernia is demonstrated.

**Conclusion:**

We showed that the laparoscopic approach can be an effective and reproducible treatment for perineal hernia.

**Learning objective:**

Understanding of the surgical steps involved in the laparoscopic repair with mesh of a recurrent perineal hernia.

## Introduction

The perineal hernia is a condition that occurs because of a defect in the pelvic diaphragm. The primary hernia is less common and affects women five times more than men due to the impairment of the pelvic floor during pregnancy. This condition is also attributable to increased intra-abdominal pressure from conditions such as obesity and ascites. The secondary hernia, which corresponds to most cases, arises due to previous pelvic surgery and is mainly associated with extensive operations such as pelvic exenteration (Stamatiou et al., 2010; Maspero et al., 2022; Cali et al., 1992; Watanabe et al., 2020). The perineal hernia is also classified into anterior or posterior according to its relationship with the superficial transverse perineal muscle. Anterior perineal hernias are located in the urogenital diaphragm surrounded by the ischiocavernosus, bulbocavernosus, and superficial transverse perineal muscles. The classic presentation is bulging in the region of the labia. Posterior perineal hernias may occur due to a defect through the muscles of the pelvic diaphragm or between the obturator internus and the iliococcygeus muscles, known as the hiatus of Schwalbe, and appear in the ischiorectal fossa. The perineal hernia usually is asymptomatic and less commonly presents with pain, dysuria, or bowel obstruction (Watanobe et al., 2020; Stamatiou et al., 2010; Cali et al., 1992).

The definitive treatment of perineal hernias is surgical repair. Many techniques have been described yet there is no gold standard treatment. The approaches include transabdominal, perineal, vaginal or a combined approach. The technique varies from primary closure of the defect or reinforcement of the pelvic floor using graft or mesh (Stamatiou et al., 2010; Cali et al., 1992).

## Patients and methods

**Figure 1:**
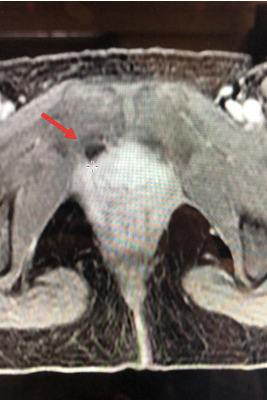
Cross section of the pelvic MRI, showing a peritoneal sac containing fat tissue herniating through the pelvic floor (red arrow).

A video presentation showing the laparoscopic repair of perineal hernia. Ethical approval was acquired and informed consent was obtained. We present the case of a parous 46-year-old woman with a prior history of a primary perineal hernia repair by laparoscopy using an absorbable mesh placed over the pelvic diaphragm muscles. She had also previously undergone bariatric surgery. 34 months after the primary perineal hernia repair, the patient presented to our department with symptomatic vulvar bulge precipitated by the squatting position. A weakness with a palpable soft mass in the right labia majora was appreciated on exam, suggestive of a recurrence of the perineal hernia. There was no sign of bowel infarction or peritoneal irritation. Pelvic magnetic resonance imaging showed a 5cm hernial sac at the right anterior pelvic wall containing adipose tissue, originating from a 1 cm defect (Figure 1).

## Results

The patient underwent a laparoscopic perineal hernia repair. Four laparoscopic ports were placed: a 10 mm infraumbilical camera port and three 5 mm ports in the lower abdomen after pneumoperitoneum was established via a Veress needle entry. A total laparoscopic hysterectomy was performed due to symptomatic adenomyosis at the beginning of the procedure. Thereafter, we turned our attention to the perineal hernia. Dissection of the space of Retzius in the direction of the lateral vaginal wall with mobilization of the bladder caudally was completed. The prevesical space was dissected and the hernial sac with adipose tissue was identified. The reduction of the hernia sac was carried out and the defect closed using 2-0 Ethibond suture. To strengthen the hernia repair, a Marlex mesh was placed across the area surrounding the hernia and attached with Tisseel, a fibrin glue, to avoid mesh migration. Peritoneal closure was performed with a running suture of 3-0 Polydioxanone.

The patient was discharged from the hospital the following day with no postoperative complications. At 6-months follow up, the patient was asymptomatic and had no signs of recurrence.

## Discussion

There is no consensus regarding the best management of anterior perineal hernias. The perineal hernia can be successfully treated with perineal, laparoscopic, or combined approach (Watanobe et al., 2020; Maspero et al., 2022). The perineal approach is traditionally the most frequently employed(Balla et al., 2017). A recent systematic review and meta- analysis included 347 cases of perineal hernia repair and showed the perineal approach as the elected in most cases (68%), followed by abdominal in 23% and combined in 4%. Out of 347 repairs, only 34 were laparoscopic (Maspero et al., 2022). The laparoscopic abdominal approach allows direct vision and, depending on the surgeon’s experience, may ensure more effective closure of the pelvic defect (Stamatiou et al., 2010; Moroni et al., 2013).

Our patient presented with a recurrent perineal hernia. Some estimates suggest this occurs in up to 50% of cases (Mack et al., 2022). Recurrence rates were 19% after perineal approach, 18% after abdominal approach, and 10% after combined approach, but no risk difference was found at meta- analysis (Maspero et al., 2022). In a systematic review of 108 patients who underwent different surgical approaches, recurrence was observed in 26 cases (24.07%). Out of these 26 recurrent cases, 7 were repeat recurrences (26.92%) (Balla et al., 2017). In the literature, the recurrence rates also vary according to the type of repair used in the abdominal approach, with findings of 16% with mesh, 20% with flap and 30% with primary repair with simple sutures (Maspero et al., 2022).

Postoperative complications of perineal hernia repair include surgical site infections, seromas, wound dehiscence, small bowel obstructions, urinary retention, and postoperative ileus. The overall postoperative complication rates were 33% after perineal or abdominal repair and 36% after mesh repair with no significant difference between biological and synthetic mesh (Maspero et al., 2022). The International Federation of Gynecology and Obstetrics (FIGO) carried out a review regarding the use of synthetic mesh for treatment of pelvic organ prolapse with controversial position statements, since most cases could be successfully treated with native tissue repair (Ugianskiene et al., 2019). Caution with the use of vaginal mesh is due to concerns about complications such as mesh erosion and exposure, sexual dysfunction, urinary tract injury, voiding dysfunction, vaginal and pelvic pain, organ or blood vessel perforation, and the need of further surgery for the removal of the complicated mesh. Abdominal mesh placement may result in lower complication rates when compared with transvaginal implantation (Ugianskiene et al., 2019). However, regarding mesh erosion the rates were not different comparing these two approaches in a systematic review of 9077 patients (MacCraith et al., 2020). Transvaginal mesh implantation should therefore be reserved for high-risk cases such as recurrent pelvic organ prolapse, those with increased intra-abdominal pressure or patients with contraindication to abdominal surgery (Ugianskiene et al., 2019). In our case of recurrent perineal hernia, we used a synthetic mesh, and we did not have any complications in 6-months follow-up.

## Conclusions

Laparoscopic repair of a perineal hernia can be an effective and safe treatment due to the advantages of minimally invasive surgery. To our knowledge, this is the first demonstration in the literature of the laparoscopic approach

## Video scan (read QR)

https://vimeo.com/772032356/3acab5cbb9

**Figure:**